# Estimating the Bioaccumulation Potential of Hydrophobic Ultraviolet Stabilizers Using Experimental Partitioning Properties

**DOI:** 10.3390/ijerph19073989

**Published:** 2022-03-27

**Authors:** Anh T. Ngoc Do, Yoonsub Kim, Yeonjeong Ha, Jung-Hwan Kwon

**Affiliations:** 1 Division of Environmental Science and Ecological Engineering, Korea University, 145 Anam-ro, Seongbuk-gu, Seoul 02841, Korea; anhdn195@korea.ac.kr (A.T.N.D.); kys0437@korea.ac.kr (Y.K.); gbhyjyh@korea.ac.kr (Y.H.); 2 Environment & Safety Research Center, Samsung Electronics Co., Ltd., Samsungjeonja-ro 1, Hwaseong-si 18448, Korea

**Keywords:** *n*-octanol/water partition constant (K_ow_), lipid/water partition constant (K_lipw_), passive dosing, UV stabilizers, bioaccumulation, fate and distribution, risk assessment

## Abstract

Although hydrophobic ultraviolet (UV) stabilizers are an emerging environmental concern because of their widespread occurrence, persistence, and bioaccumulation potential, experimental values of their partitioning properties required for risk assessment are scarce. In this study, *n*-octanol-water partition (K_ow_) and lipid–water partition constants (K_lipw_), which are key parameters for environmental risk assessment, were experimentally determined for five selected hydrophobic UV stabilizers (UV326, UV327, UV328, UV329, and UV531) based on third-phase partitioning among polydimethylsiloxane (PDMS), water, and *n*-octanol/lipid. The partition constants between PDMS and water (K_PDMSw_), obtained using the dynamic permeation method were used to derive K_ow_ and K_lipw_. The obtained log K_ow_ and log K_lipw_ values were in the ranges of 7.08–7.94 and 7.50–8.34, respectively, indicating that the UV stabilizers exhibited a high bioaccumulation potential in aquatic environments. The experimental K_ow_ and K_lipw_ values obtained in this study provide valuable information for the evaluation of the fate, distribution, bioavailability, and toxicity of the UV stabilizers in aquatic environments.

## 1. Introduction

Ultraviolet (UV) stabilizers have been widely used in personal care products, as additives in polymeric food-contacting materials, and as surface coatings [1,2]. Benzotriazoles and benzophenones are among the most commonly used UV stabilizers [3]. These UV stabilizers are aromatic and highly hydrophobic molecules that are of emerging environmental concern owing to their widespread occurrence, persistence, bioaccumulation potential, and toxicity [4,5]. With the increasing production and consumption of UV stabilizers, their residues have been found in humans (e.g., breast milk and urine) as well as various environmental media (e.g., coastal environments or house dust) [5,6,7,8,9,10,11,12,13,14]. Recently, 2.4-di-*tert*-butyl-6-(5-chloro-2H-benzotriazol-2-yl)phenol (UV327) and 2-(2H-benzotriazol-2-yl)-4.6-bis(2-methyl-2-butanyl)phenol (UV328) have been listed as substances of very high concern (SVHC) under REACH (Registration, Evaluation, and Authorization of Chemicals) owing to their high potential for bioaccumulation, persistence, and toxicity [15], and (2-hydroxy-4-octoxyphenyl) phenylmethanone (UV531) has been included in the European Community Rolling Action Plan (CoRAP) for evaluation in the upcoming years [16]. Although these benzotriazoles and benzophenones have received considerable attention from researchers and policymakers, it is challenging to experimentally obtain fundamental parameters for risk assessment, such as *n*-octanol-water partition (K_ow_) and lipid–water partition constants (K_lipw_), owing to their strong hydrophobicity.

K_ow_ is the most widely used parameter for predicting the environmental distribution and potential bioaccumulation of persistent organic pollutants (POPs) [17]. However, previous studies have shown thermodynamic differences between the *n*-octanol–water partitioning system and the actual biological uptake because the interactions of pollutants with highly organized lipid membranes are different from those with bulky octanol solvents [17,18]. To evaluate the fate and behavior of hydrophobic organic chemicals (HOCs) in aquatic environments more precisely, intermediate lipid–water systems have recently been used in pharmacology and environmental toxicology to assess the bioconcentration of organic compounds. Only a few experimental partition constants have been reported for storage lipids, which are the major class of lipids in living organisms [19]. Therefore, to assess the bioaccumulation potential of UV stabilizers in aquatic environments, it is essential to obtain both K_ow_ and K_lipw_ values.

UV stabilizers (i.e., UV328, UV329, and UV531) are classified as high-production-volume chemicals (>1000 metric tons per producer/importer annually) [20], and their physicochemical properties required for registration have been measured and submitted [21]. However, experimental K_ow_ values have been reported for a few hydrophobic UV stabilizers using the HPLC method (database is available in OECD QSAR Toolbox 4.4.1—https://qsartoolbox.org, accessed on 11 February 2022) [22], which might be limited for highly hydrophobic chemicals (log K_ow_ > 6) owing to the limited number of highly hydrophobic substances used for the calibration of the method. Furthermore, no K_lipw_ values have been reported for UV stabilizers. Experimental methods have been developed for “difficult-to-test” compounds, such as highly hydrophobic UV stabilizers [23,24]. The partition constants for highly hydrophobic organic chemicals can be estimated using high-performance liquid chromatography (HPLC) [25]; kinetic methods, such as the dynamic permeation method [24]; or the third-phase partitioning method [26]. Thus, precisely measured values of K_ow_ and K_lipw_ should greatly help to improve evaluations of the distribution, bioavailability, exposure, and toxicity of UV stabilizers in aquatic environments [27].

The aim of this study was to experimentally determine K_ow_ and K_lipw_ values of five widely used UV stabilizers: 2-(5-chloro-2H-benzotriazol-2-yl)-4-methyl-6-(2-methyl-2-propanyl) phenol (UV 326), UV 327, UV 328, 2-(2H-benzotriazol-2-yl)-4-(1,1,3,3-tetramethylbutyl)phenol (UV329), and UV531. The distribution constants of the selected UV stabilizers between polydimethylsiloxane (PDMS) and water (K_PDMSw_) were obtained using the dynamic permeation method. Using K_PDMSw_ values, K_ow_ and K_lipw_ values were determined using the third-phase partitioning method. The experimentally determined log K_ow_ and K_lipw_ values were then compared with available estimated values in the literature and used to critically discuss the fate of UV stabilizers and their accumulation in aquatic environments.

## 2. Materials and Methods

### 2.1. Materials and Chemicals

The five UV stabilizers, namely UV326 (>98%), UV327 (>98%), UV328 (>98%), UV329 (>98%), and UV531 (>98%), were purchased from Sigma-Aldrich (St. Louis, MO, USA). Detailed information regarding the five target chemicals is presented in Table 1. Fish oil from menhaden was purchased from Sigma-Aldrich. All solvents (methanol, acetonitrile, and water) used in this study were of analytical grade. Medical-grade polydimethylsiloxane (PDMS) sheets (thickness = 1 mm, density = 1170 kg m^−3^) were purchased from Specialty Silicone Products, Inc. (Ballston Spa, NY, USA). The PDMS sheets were cut into disks (diameter = 6 mm) to determine the partition constants. The custom-cut PDMS disks and sheets were cleaned using *n*-hexane, followed by methanol for 2 h each and stored in methanol until use.

### 2.2. Partition Constants between PDMS and Water (K_PDMSw_)

K_PDMSw_ values were measured using the aqueous boundary layer (ABL) permeation method. The theory and experimental procedure for this method have been described in detail in existing literature [24,26]. Briefly, by neglecting the mass loss in the aqueous solution between the two PDMS disks because of the high holding capacity of PDMS for HOCs, the concentration in the acceptor disk (C_acceptorPDMS_) is given by Equation (1), where C_0_ (mM) is the initial concentration of chemicals in the donor PDMS disk and k (s^−1^) is the mass transfer-rate constant through the ABL.

(1) $$C_{\mathrm{acceptorPDMS}}=\frac{C_{0}}{2}\left[1-exp\left(-kt\right)\right]$$

(2) $$\ln\left(1-\frac{2C_{\mathrm{acceptorPDMS}}}{C_{0}}\right)=-kt$$

**Figure 1:**
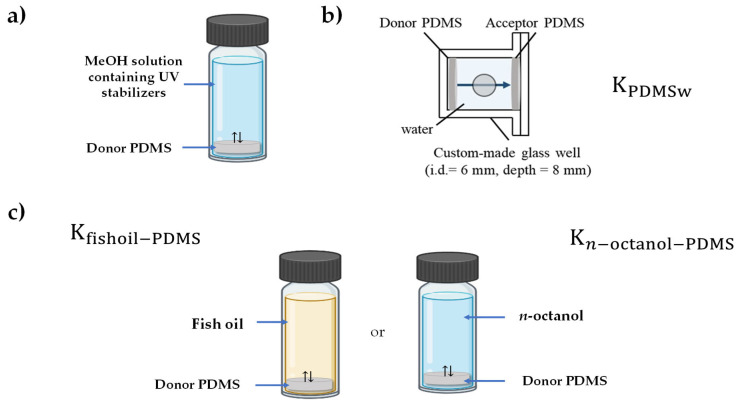
Schematic diagram of the measurement of the partition constants of the five UV stabilizers. (**a**) Loading selected UV stabilizers from MeOH solution to donor PDMS; (**b**) partition constants between PDMS and water (K_PDMSw_) using the aqueous boundary layer-permeation method [24]; (**c**) determination of *n*-octanol-PDMS and fish oil-PDMS partition constants (K_n-octanol-PDMS_ and K_fishoil-PDMS_).

Equation (2) can be obtained by rearranging Equation (1), and the mass transfer-rate constant (k) can be determined using a linear regression between $\ln\left(1-\frac{2C_{\mathrm{acceptorPDMS}}}{C_{0}}\right)$ and *t*. Mass transfer resistance in aqueous solution is dominant for highly hydrophobic chemicals because PDMS is an amorphous polymeric material. Thus, K_PDMSw_ (L_w_ L_PDMS_^−1^) can be derived using Equation (3), and the aqueous diffusion coefficient (*D_w_* in m^2^ s^−1^) can be estimated from the relationship with the molar mass of the five UV stabilizers using Equation (4) [24].

(3) $$k\approx \frac{D_{w}}{K_{PDMSw}\delta_{w}}\frac{A_{PDMS}}{V_{PDMS}}$$

(4) $$D_{w}\left(m^{2}\cdot s^{-1}\right)=\frac{2.7\times 10^{-8}}{MW^{0.71}}$$

where *δ_w_* is the thickness of the aqueous boundary layer (m), *A_PDMS_* is the surface area of PDMS (m^2^), *V_PDMS_* is the volume of PDMS (m^3^), and *MW* is the molecular weight of the chemical (g mol^−1^). A donor PDMS disk (diameter = 6 mm, thickness = 1 mm) was loaded with each UV stabilizer using methanol as the solvent for 24 h (Figure 1a). Donor and acceptor PDMS disks were placed at opposite ends of a custom-made glass well filled with water. The aqueous solution between the two disks was agitated with a stainless steel disk (diameter = 5.08 mm, thickness = 0.635 mm) at 300 rpm using a VP710F tumble stirrer (V&P Scientific Inc., San Diego, CA, USA) (Figure 1b). The thickness of the ABL was set to 12.5 μm because the experimental system used in this study was the same as that used in previous studies [24,26]. After a designated time, the disks were removed and rinsed with 1 mL methanol:water (6:4, *v*/*v*) and 1 mL water. The UV stabilizers in the disks were extracted using 1 mL acetonitrile (ACN) for 24 h. The mass transfer rate was calculated by measuring the changes in the concentrations of both donor and acceptor disks (Equation (2)). K_PDMSw_ was derived using the mass transfer-rate equation (Equation (3)). All experiments were conducted at 25 °C. A schematic diagram for determining K_PDMSw_ is shown in Figure 1a,b.

### 2.3. n-Octanol-Water and Fish Oil–Water Partition Constants

The partition constants between *n*-octanol or fish oil and PDMS (K_octanol-PDMS_ or K_fish oil-PDMS_) were obtained by measuring the equilibrium concentrations of *n*-octanol or fish oil and PDMS in a batch system. Then, the K_octanol-PDMS_ or K_fish oil-PDMS_ values were used to calculate K_ow_ or K_lipw_, assuming that the activity coefficients in PDMS were the same in water and in *n*-octanol or fish oil. A PDMS disk (diameter = 10 mm, thickness = 1 mm) was loaded with each UV stabilizer, dissolved in a 500 or 1000 mg L^−1^ methanol, for 24 h (Figure 1a). The loaded PDMS disks were rinsed with 1 mL methanol:water (6:4, *v*/*v*) and 1 mL water and placed in vials containing 125, 166, and 250 μL of *n*-octanol or fish oil (Figure 1c). Each vial was agitated at 25 °C and 150 rpm in the dark in a shaking incubator. Preliminary experiments showed that 24 h and 48 h were sufficient to attain equilibrium in the octanol–PDMS and fish oil–PDMS systems, respectively. After equilibrium was established, the PDMS disk was collected from the vial, rinsed using 1 mL methanol:water (6:4, *v*/*v*) and 1 mL water, and extracted using 1 mL ACN. Fish oil and *n*-octanol were diluted in ACN. The values of K_octanol-PDMS_ or K_fish oil-PDMS_ were obtained using a linear regression between the concentration in *n*-octanol or fish oil and that in PDMS. Finally, the values of K_ow_ and K_lipw_ were calculated using the third-phase equilibrium method using Equations (5) and (6):

(5) $$K_{ow}=K_{PDMSw}\times K_{octanal\text{-}PDMS}$$

(6) $$K_{lipw}=K_{PDMSw}\times K_{fishoil\text{-}PDMS}$$

### 2.4. Instrumental Analyses

The concentrations of the five tested chemicals were quantified using a Waters ACQUITY ultra performance liquid chromatograph (UPLC) with a photodiode array (PDA) detector. The absorbance was measured at 221 nm (UV326), 204 nm (UV327 and UV328), 218 nm (UV329), and 287 nm (UV531). The UV stabilizers were separated using a C18 column (2.1 mm × 50 mm, 1.7 μm, Waters) at 35 °C. The mobile phase comprised 95% acetonitrile and 5% water in isocratic mode with a flow rate of 0.2 mL min^−1^.

## 3. Results

**Figure 2:**
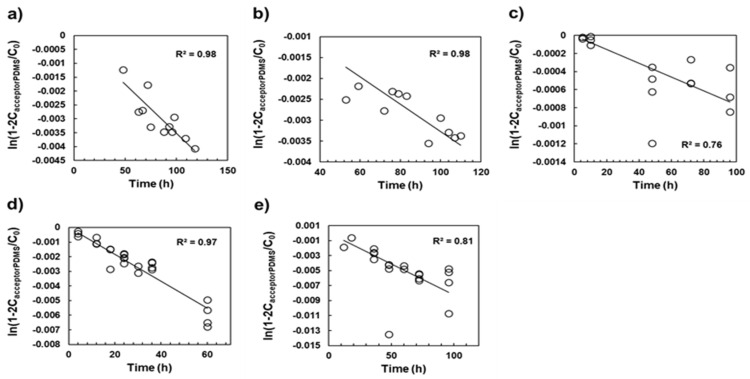
Mass transfer kinetics of (**a**) UV326, (**b**) UV327, (**c**) UV328, (**d**) UV329, and (**e**) UV531 for the determination of K_PDMSw_ using the aqueous boundary layer (ABL) permeation method. The solid lines denote regression calculated using Equation (2).

Figure 2 shows the mass transfer kinetics of the selected UV stabilizers from the donor PDMS to the acceptor PDMS for the determination of K_PDMSw_. The desorption rate constants (k) for the five test chemicals were obtained using Equation (2). Except for data for UV531 (R^2^ = 0.81) and UV328 (R^2^ = 0.76), the experimental data fit well (R^2^ > 0.9). The K_PDMSw_ values were then calculated using Equation (3), and log K_PDMSw_ ranged from 6.14 to 7.22, and the uncertainties of the K_PDMSw_ were calculated as the 95% upper and lower limits of the error propagation from the standard error of regression and the estimated uncertainties of the thickness of the ABL (Table 2). These values were used to estimate the K_ow_ and K_lipw_ values.

**Figure 3:**
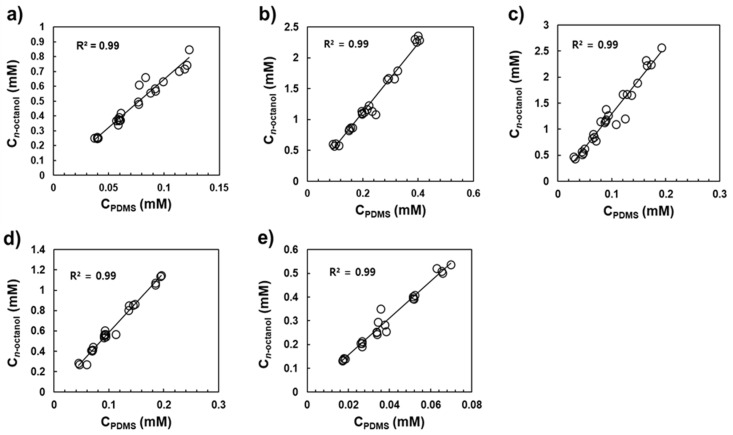
Regression between *n*-octanol and PDMS of (**a**) UV326, (**b**) UV327, (**c**) UV328, (**d**) UV329, and (**e**) UV531. The solid lines denote linear regression lines.

**Figure 4:**
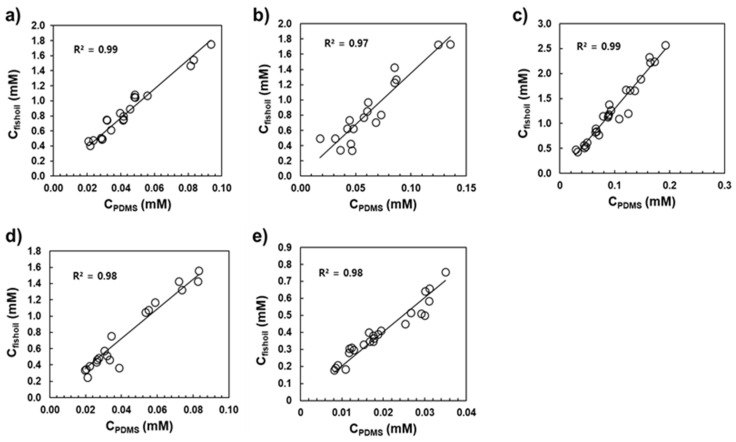
Regression between fish oil and PDMS of (**a**) UV326, (**b**) UV327, (**c**) dUV328, (**d**) UV329, and (**e**) UV531. The solid lines denote linear regression lines.

The values of K_ow_ and K_lipw_ were obtained from the slopes of the linear regression between the concentration in *n*-octanol or fish oil and that in PDMS (Figure 3 and Figure 4). As shown in Figure 3 and Figure 4, good linear relationships were observed for the five UV stabilizers within the concentration range investigated (R^2^ > 0.97). The resulting values of log K_ow_ and log K_lipw_, calculated using Equations (5) and (6), were in the ranges of 7.08–7.94 and 7.50–8.34, respectively (Table 2). As shown in Table 2, log K_lipw_ values were consistently greater than the log K_ow_ values by 0.38–0.49 log units. Table 2 presents the literature values of log K_ow_ submitted to the European Chemical Agency (ECHA) using the HPLC retention time method [29] and those estimated using EPI Suite version 4.11 [30] and the ALOGPS 2.1 program [31]. It should be noted that the K_ow_ and K_lipw_ values in this study were measured at 25 °C, the standard thermodynamic temperature for providing reference values. Further studies on the effects of environmental factors (e.g., temperature and electrolytes) on K_ow_ and K_lipw_ values would extend the applicability of those partition constants under various environmental conditions [19,32].

## 4. Discussion

The experimental values of K_ow_ and K_lipw_ can be obtain by using direct-measurement methods (e.g., slow-stirring, shake-flask, and generator column method) or indirect methods (e.g., reverse-phase high-performance liquid chromatography). However, shake-flask and generator column method and reverse-phase high-performance liquid chromatography might be not appropriate for determining of K_ow_ and K_lipw_ for highly hydrophobic compounds (log K_ow_ > 6) [17,33]. The slow-stirring method has been reported in literature for measuring the K_ow_ and K_lipw_ for highly hydrophobic chemicals; however, the time required to reach to equilibrium generally takes from a few days up to a few weeks [17,34,35]. To overcome the difficulties in measuring the K_ow_ and K_lipw_ for highly hydrophobic chemicals (i.e., time-consuming, extremely low water solubility, degradation of parent compound during experiment period), the third-phase method, in which a polymer serve as a reference partitioning phase, was proposed to determine the K_ow_ and K_lipw_ for hydrophobic chemicals with log K_ow_ > 6 [36,37]. As shown in Table 2, the log K_ow_ values of the five tested UV stabilizers varied substantially depending on the experimental and estimation methods. The experimental log K_ow_ data for the UV stabilizers have been as yet reported in literature using a HPLC-based method [29]. The experimental log K_ow_ values in this study were consistently higher and could be more accurate than those reported in the registration dossier to ECHA [29], which reported only lower bounds estimated using the HPLC retention time (Table 2). Because the HPLC retention times of chemicals with experimental log K_ow_ values between 0 and 6 were used in the calibration of the method (OECD Guideline 117), greater uncertainties resulted when the log K_ow_ values were above 6. However, for highly hydrophobic substances, log K_ow_ values are often estimated using quantitative structure–property relationship (QSPR) methods (e.g., ALOGPS, KOWWIN) (Table 2). Although the predicted log K_ow_ values by KOWWIN or ALOGPS are lower than the experimental values in this study, the prediction for K_ow_ by the QSPR methods agree quite well with experimental data in this case, especially by KOWWIN (the difference within 1 log unit, except for UV326). The predicted log K_ow_ might not be accurate because the predicted K_ow_ values from the commercial software packages are derived from the experimental K_ow_ values of their nearest analogs [38,39]. An important additional reason here is that at least UV-328 can make an intramolecular hydrogen bond that is not captured in EPISuite [40]. Therefore, the lack of measured data for highly hydrophobic chemicals leads to the inevitable accuracy of the QSPR methods [39]. Although there are certain limitations, such as the long experimental time, high cost, and expensive instruments to detect trace concentrations of analyte to obtain reliable K_ow_ values, these experimental methods are highly recommended for evaluation of UV stabilizers. *Ab initio* methods are also used as an alternative to predict partition constants for HOCs because they do not rely on the quality of experimental data in the training set. A commonly used program from this is the COSMOtherm program, which is based on quantum chemical descriptors [41,42]. Interestingly, the log K_ow_ of UV327 and UV328 in this study agrees well with estimations made using COSMOtherm (log K_ow_ of UV327 and UV328 are 7.91 and 8.5, respectively [40,43]. The difference between the predicted values by COSMOtherm and the experimental data for log K_ow_ is within 0.6 log unit, supporting the usefulness of these *ab initio* methods for “difficult-to-test” substances.

In this study, the K_lipw_ values were generally higher than the K_ow_ values, which can be explained by the fact that lipid storage has a highly organized structure compared to the less organized structure of *n*-octanol, and the driving forces for partitioning into saturated lipid storage can be differentiated from those for *n*-octanol [18]. There have been studies that experimentally determined lipid–water partitioning constants for a broad range of compounds, including ionic substances, strongly hydrophobic chemicals, and nanomaterials, via different methods, such as potentiometry, the equilibrium dialysis technique, and methods using solid-supported lipid membranes [18,44,45]. To the best of our knowledge, this is the first report of the K_lipw_ values of the selected UV stabilizers. Although this study used a storage lipid (e.g., fish oil) as a representative lipid material, the lipid accumulation properties of chemicals in storage lipids might differ from those in membrane lipids (i.e., phospholipids). Previous studies have demonstrated that the K_lipw_ values of hydrophobic chemicals toward storage lipids were similar to those of hydrophobic chemicals toward a model membrane (i.e., the difference was less than 1 log unit) [19,46]. In addition, storage lipids from different origins (i.e., olive oil, milk fat, fish oil, goose oil, and soybean oil) do not differ in their accumulation properties for various polar and nonpolar organic chemicals [19]. Thus, the K_lipw_ values in this study are good indicators of the biopartitioning of UV stabilizers. For highly hydrophobic organic chemicals, the time required to attain a steady state is often longer than the lifespan of aquatic species, necessitating the consideration of other kinetic parameters, such as growth and metabolic transformation rates, to obtain the bioconcentration factor [47]. Bioaccumulation of UV stabilizers occurs primarily after their ingestion by organisms. There is evidence of the bioaccumulation of UV328 in fish, crustaceans, marine mammals, and algae [48]. Furthermore, biomagnification of UV531 was observed by Peng et al., and the lipid normalized concentration of UV531 in mantis shrimp was higher than that in its prey, sword and Kurumu prawns [49]. In another study, UV326, UV328, and UV329 were found at concentrations ranging from 1.34 to 45.6 ng g^−1^ (dry weight, dw) on Gran Canaria Island (Spain) [50]. Hasegawa et al. recently showed similar accumulation patterns between direct ingestion from the water column and indirect exposure via the trophic transfer pathway for UV327 in marine fish [51]. In a recent monitoring study, the sum of nine benzotriazole UV stabilizers (including the five target chemicals in this study) were found in river water (17.0–32.5 ng L^−1^) and sediment (2.0–22.6 ng g^−1^, dw) in Korea [52]. Furthermore, the benzotriazole (UV326, UV327, UV328, and UV329) bioaccumulation in crucian carp muscle ranged from 0.345 to 5.94 (ng g^−1^, wet weight), with UV329 being the dominant compound in all crucian carp. The field-derived log bioaccumulation factor (BAF) and biota-sediment accumulation factor (BSAF) for UV329 and UV326 were also calculated during 2018–2020 as 2.89, 2.67, −0.02, and −0.02, respectively [52].

These observations were consistent with the extremely high log K_ow_ and log K_lipw_ values obtained in this study. However, the log BAF and estimated partition coefficients between sediment and water were lower than those estimated from log K_ow_ and log K_lipw_. As previously mentioned, competition between partitioning processes and other processes may explain these differences. In addition, there are other aspects to be considered for a better understanding of the environmental behavior of UV stabilizers because large amounts of UV stabilizers are used as plastic additives, and they are likely to leach very slowly from these plastics [53]. As microplastics have long environmental residence time [54], they may contribute to the long-range transport of UV stabilizers in the oceans. Because UV stabilizers do not readily biodegrade [43,55], the evaluation of their long-range transport and bioaccumulation potential is important to determine whether they should be listed as POPs. Therefore, future studies on their plastic-mediated, long-range transport as well as bioaccumulation in aquatic species are required. The experimental partitioning properties reported in this study are of considerable value for the evaluation of environmental flux by calculating fugacities in different media [56].

## 5. Conclusions

In this study, *n*-octanol-water partition (K_ow_) and lipid–water partition (K_lipw_) constants were experimentally measured to estimate the bioaccumulation of five UV stabilizers in aquatic environments. The partition constants between polydimethylsiloxane (PDMS) and water (K_PDMSw_) of UV stabilizers were obtained using the dynamic permeation method. Consequently, the partition constants between octanol and water (K_ow_) and lipid and water (K_fishoil-water_) were determined using the third-phase equilibrium method. The results showed that the values of log K_ow_ and log K_lipw_ were in the ranges of 7.08–7.94 and 7.50–8.34, respectively, indicating the high potential bioaccumulation of the five UV stabilizers in aquatic environments. The experimental log K_ow_ and log K_lipw_ values also provide valuable information for risks assessment of UV stabilizers.

## Figures and Tables

**Table 1:** Names, molecular structures, molecular weights, empirical formulas, and CAS numbers of UV326, UV327, UV328, UV329, and UV531.

Compounds	Molecular Structure	Molecular Weight (g mol^−1^)	Empirical Formula	CAS No.
UV326	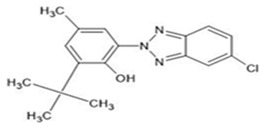	315.80	C_17_H_18_ClN_3_O	3896-11-5
UV327	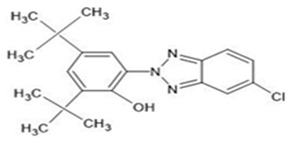	357.88	C_20_H_24_ClN_3_O	3864-99-1
UV328	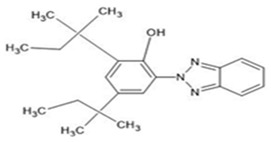	351.49	C_22_H_29_N_3_O	25973-55-1
UV329	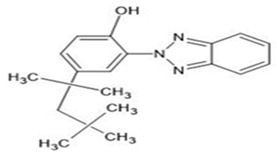	323.43	C_20_H_25_N_3_O	3147-75-9
UV531	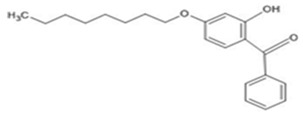	326.436	C_21_H_26_O_3_	1843-05-6

**Table 2:** Values of D_w_, log K_PDMSw_, log K_lipw_, and log K_ow_ (experimentally determined and model predicted values) of the five UV stabilizers.

Chemicals	D_w_^a^ (m^2^·s^−1^)	Experimental Data				Predicted Data	
		log K_PDMSw_ (K_PDMSw_ in L_w_·L_PDMS_^−1^)	log K_lipw_ (K_lipw_ in L_w_·L_lip_^−1^)	log K_ow_ (K_ow_ in L_w_·L_o_^−1^)	log K_ow_ (ECHA) ^b^	log K_ow_ (EPI Suite) ^c^	log K_ow_ (ALOGPS) ^d^
UV326	4.54 × 10^−10^	6.57 (6.53, 6.62)	7.86 (7.81, 7.91)	7.38 (7.34, 7.43)	>6.5 (23 °C, pH 6.4)	5.55	5.70
UV327	4.15 × 10^−10^	6.56 (6.53, 6.60)	7.69 (7.75, 7.64)	7.31 (7.27, 7.35)	—	6.91	6.27
UV328	4.21 × 10^−10^	7.22 (7.13, 7.33)	8.34 (8.24, 8.45)	7.94 (7.85, 8.05)	>6.5 (23 °C, pH 6.4)	7.25	6.54
UV329	4.46 × 10^−10^	6.14 (6.06, 6.20)	7.40 (7.34, 7.46)	6.91 (6.86, 6.97)	>6.5 (23 °C, pH 6.4)	6.21	5.83
UV531	4.43 × 10^−10^	6.19 (6.07, 6.38)	7.50 (7.37, 7.68)	7.08 (6.96, 7.27)	—	6.96	6.12

Values in parentheses are the lower and upper 95% confidence intervals. The confidence limits of K_lipw_ and K_ow_ were calculated using error propagation. ^a^ D_w_ values are calculated using Equation (4) [28]. ^b^ the partition coefficient was measured using HPLC. OECD TG 117, Registration Dossier, ECHA [29]. ^c^ Estimated values from EPI Suite version 4.11 [30]. ^d^ Estimated values from the ALOGPS 2.1 program [31].

## Data Availability

The data presented in this study are available on request.

## References

[1] Kwon J.H., Chang S., Hong S.H., Shim W.J. (2017). Microplastics as a vector of hydrophobic contaminants: Importance of hydrophobic additives. Integr. Environ. Assess. Manag..

[2] Pfaendner R. (2006). How will additives shape the future of plastics?. Polym. Degrad. Stab..

[3] Zhang Z., Ren N., Li Y.-F., Kunisue T., Gao D., Kannan K. (2011). Determination of benzotriazole and benzophenone UV filters in sediment and sewage sludge. Environ. Sci. Technol..

[4] Nakata H., Shinohara R.-I., Nakazawa Y., Isobe T., Sudaryanto A., Subramanian A., Tanabe S., Zakaria M.P., Zheng G.J., Lam P.K. (2012). Asia–Pacific mussel watch for emerging pollutants: Distribution of synthetic musks and benzotriazole UV stabilizers in Asian and US coastal waters. Mar. Pollut. Bull..

[5] Fent K., Chew G., Li J., Gomez E. (2014). Benzotriazole UV-stabilizers and benzotriazole: Antiandrogenic activity in vitro and activation of aryl hydrocarbon receptor pathway in zebrafish eleuthero-embryos. Sci. Total Environ..

[6] Apel C., Tang J., Ebinghaus R. (2018). Environmental occurrence and distribution of organic UV stabilizers and UV filters in the sediment of Chinese Bohai and Yellow Seas. Environ. Pollut..

[7] García-Guerra R.B., Montesdeoca-Esponda S., Sosa-Ferrera Z., Kabir A., Furton K.G., Santana-Rodríguez J.J. (2016). Rapid monitoring of residual UV-stabilizers in seawater samples from beaches using fabric phase sorptive extraction and UHPLC-MS/MS. Chemosphere.

[8] Kim J.-W., Chang K.-H., Prudente M., Viet P.H., Takahashi S., Tanabe S., Kunisue T., Isobe T. (2019). Occurrence of benzotriazole ultraviolet stabilizers (BUVSs) in human breast milk from three Asian countries. Sci. Total Environ..

[9] Langford K.H., Reid M.J., Fjeld E., Øxnevad S., Thomas K.V. (2015). Environmental occurrence and risk of organic UV filters and stabilizers in multiple matrices in Norway. Environ. Int..

[10] Lu Z., de Silva A.O., Provencher J.F., Mallory M.L., Kirk J.L., Houde M., Stewart C., Braune B.M., Avery-Gomm S., Muir D.C. (2019). Occurrence of substituted diphenylamine antioxidants and benzotriazole UV stabilizers in Arctic seabirds and seals. Sci. Total Environ..

[11] Montesdeoca-Esponda S., Álvarez-Raya C., Torres-Padrón M.E., Sosa-Ferrera Z., Santana-Rodríguez J.J. (2019). Monitoring and environmental risk assessment of benzotriazole UV stabilizers in the sewage and coastal environment of Gran Canaria (Canary Islands, Spain). J. Environ. Manag..

[12] Montesdeoca-Esponda S., Vega-Morales T., Sosa-Ferrera Z., Santana-Rodríguez J. (2013). Extraction and determination methodologies for benzotriazole UV stabilizers in personal-care products in environmental and biological samples. Trends Analyt. Chem..

[13] Wick A., Jacobs B., Kunkel U., Heininger P., Ternes T.A. (2016). Benzotriazole UV stabilizers in sediments, suspended particulate matter and fish of German rivers: New insights into occurrence, time trends and persistency. Environ. Pollut..

[14] Apel C.H. (2019). Organic UV Stabilizers in the Coastal and Marine Environment: European North and Baltic Seas Compared to Chinese Bohai and Yellow Seas.

[15] ECHA (2022). Candidate List of Substances of Very High Concern for Authorisation. https://echa.europa.eu/web/guest/candidate-list-table.

[16] ECHA (2022). Community Rolling Action Plan (CoRAP): List of Substances. https://echa.europa.eu/information-on-chemicals/evaluation/community-rolling-action-plan/corap-table.

[17] Jabusch T.W., Swackhamer D.L. (2005). Partitioning of polychlorinated biphenyls in octanol/water, triolein/water, and membrane/water systems. Chemosphere.

[18] Kwon J.H., Liljestrand H.M., Katz L.E. (2006). Partitioning of moderately hydrophobic endocrine disruptors between water and synthetic membrane vesicles. Environ. Toxicol. Chem. Int. J..

[19] Geisler A., Endo S., Goss K.-U. (2012). Partitioning of organic chemicals to storage lipids: Elucidating the dependence on fatty acid composition and temperature. Environ. Sci. Technol..

[20] OECD (2022). HPV Database. https://hpvchemicals.oecd.org/UI/Search.aspx.

[21] OECD (2004). The 2004 OECD List of High Production Volume Chemicals.

[22] (2020). OECD, QSAR Toolbox 4.4.1 (software). https://qsartoolbox.org.

[23] Birch H., Redman A.D., Letinski D.J., Lyon D.Y., Mayer P. (2019). Determining the water solubility of difficult-to-test substances: A tutorial review. Anal. Chim. Acta.

[24] Kwon J.-H., Wuethrich T., Mayer P., Escher B.I. (2007). Dynamic permeation method to determine partition coefficients of highly hydrophobic chemicals between poly (dimethylsiloxane) and water. Anal. Chem..

[25] Valkó K. (2004). Application of high-performance liquid chromatography based measurements of lipophilicity to model biological distribution. J. Chromatogr. A.

[26] Lee H., Shim W.J., Kwon J.H. (2014). Sorption capacity of plastic debris for hydrophobic organic chemicals. Sci. Total Environ..

[27] Groh K.J., Backhaus T., Carney-Almroth B., Geueke B., Inostroza P.A., Lennquist A., Leslie H.A., Maffini M., Slunge D., Trasande L. (2019). Overview of known plastic packaging-associated chemicals and their hazards. Sci. Total Environ..

[28] Schwarzenbach R.P., Gschwend P.M., Imboden D.M. (2016). Environmental Organic Chemistry.

[29] ECHA (2022). Registration Dossier. https://echa.europa.eu/regulations/reach/substance-registration/the-registration-dossier.

[30] EPA (2012). Exposure Assessment Tools and Models, Estimation Program Interface (EPI) Suite, V 4.11.

[31] VCCL (2022). ALOGPS 2.1 Program. http://www.vcclab.org.

[32] Wille S., Buggert M., Mokrushina L., Arlt W., Smirnova I. (2010). Effect of Electrolytes on Octanol-Water Partition Coefficients: Calculations with COSMO-RS. Chem. Eng. Technol..

[33] Van Leeuwen C.J., Vermeire T.G. (2007). Risk Assessment of Chemicals: An Introduction.

[34] Jonker M.T. (2016). Determining octanol–water partition coefficients for extremely hydrophobic chemicals by combining “slow stirring” and solid-phase microextraction. Environ. Toxicol. Chem..

[35] Tolls J., Bodo K., de Felip E., Dujardin R., Kim Y.H., Moeller-Jensen L., Mullee D., Nakajima A., Paschke A., Pawliczek J.B. (2003). Slow-Stirring method for determining the n-octanol/water partition coefficient (pow) for highly hydrophobic chemicals: Performance evaluation in a ring test. Environ. Toxicol. Chem. Int. J..

[36] Endo S., Mewburn B., Escher B.I. (2013). Liposome and protein–water partitioning of polybrominated diphenyl ethers (PBDEs). Chemosphere.

[37] Gilbert D., Witt G., Smedes F., Mayer P. (2016). Polymers as reference partitioning phase: Polymer calibration for an analytically operational approach to quantify multimedia phase partitioning. Anal. Chem..

[38] Mannhold R., Poda G.I., Ostermann C., Tetko I.V. (2009). Calculation of molecular lipophilicity: State-of-the-art and comparison of log P methods on more than 96,000 compounds. J. Pharm. Sci..

[39] Hanson K.B., Hoff D.J., Lahren T.J., Mount D.R., Squillace A.J., Burkhard L.P. (2019). Estimating n-octanol-water partition coefficients for neutral highly hydrophobic chemicals using measured n-butanol-water partition coefficients. Chemosphere.

[40] ECHA (2021). UV-328. Draft Risk Profile. https://echa.europa.eu/documents/10162/c0604545-a115-9c61-a2ec-fefa5bdc5880.

[41] Stenzel A., Goss K.U., Endo S. (2014). Prediction of partition coefficients for complex environmental contaminants: Validation of COSMOtherm, ABSOLV, and SPARC. Environ. Toxicol. Chem..

[42] Wang Z., MacLeod M., Cousins I.T., Scheringer M., Hungerbühler K. (2011). Using COSMOtherm to predict physicochemical properties of poly-and perfluorinated alkyl substances (PFASs). Environ. Chem..

[43] ECHA (2015). Support Document for Identification of 2,4-di-tert-butyl-6-(5-chlorobenzotriazol-2-yl)phenol (UV-327) as a Substance of Very High Concern Because of Its vPvB (Article 57 e) Properties. https://echa.europa.eu/documents/10162/5d71b975-bba0-482d-9a5e-a102c7a0fc0d.

[44] Escher B.I., Schwarzenbach R.P., Westall J.C. (2000). Evaluation of liposome−water partitioning of organic acids and bases. 2. Comparison of experimental determination methods. Environ. Sci. Technol..

[45] Ha Y., Katz L.E., Liljestrand H.M. (2015). Distribution of fullerene nanoparticles between water and solid supported lipid membranes: Thermodynamics and effects of membrane composition on distribution. Environ. Sci. Technol..

[46] Endo S., Escher B.I., Goss K.-U. (2011). Capacities of membrane lipids to accumulate neutral organic chemicals. Environ. Sci. Technol..

[47] Nichols J.W., Huggett D.B., Arnot J.A., Fitzsimmons P.N., Cowan-Ellsberry C.E. (2013). Toward improved models for predicting bioconcentration of well-metabolized compounds by rainbow trout using measured rates of in vitro intrinsic clearance. Environ. Toxicol. Chem..

[48] Giraudo M., Cottin G., Esperanza M., Gagnon P., Silva A.O.D., Houde M. (2017). Transcriptional and cellular effects of benzotriazole UV stabilizers UV-234 and UV-328 in the freshwater invertebrates *Chlamydomonas reinhardtii* and *Daphnia magna*. Environ. Toxicol. Chem..

[49] Peng X., Fan Y., Jin J., Xiong S., Liu J., Tang C. (2017). Bioaccumulation and biomagnification of ultraviolet absorbents in marine wildlife of the Pearl River Estuarine, South China Sea. Environ. Pollut..

[50] Montesdeoca-Esponda S., Torres-Padrón M.E., Novák M., Krchová L., Sosa-Ferrera Z., Santana-Rodríguez J.J. (2020). Occurrence of benzotriazole UV stabilizers in coastal fishes. J. Environ. Manage..

[51] Hasegawa T., Mizukawa K., Yeo B.G., Sekioka T., Takada H., Nakaoka M. (2022). Trophic transfer of microplastics enhances plastic additive accumulation in fish. bioRxiv.

[52] National Institute of Environmental Research (NIER) (2020). Study on Accumulation of Emerging Contaminants in Domestic Aquatic Ecosystem (III). https://www.me.go.kr/home/file/readDownloadFile.do?fileId=128417&fileSeq=13.

[53] Do A.T.N., Ha Y., Kang H.-J., Kim J.M., Kwon J.-H. (2021). Equilibrium leaching of selected ultraviolet stabilizers from plastic products. J. Hazard. Mater..

[54] Lohmann R. (2017). Microplastics are not important for the cycling and bioaccumulation of organic pollutants in the oceans—But should microplastics be considered POPs themselves?. Integr. Environ. Assess. Manag..

[55] ECHA (2014). Support Document for Identification of 2-(2H-benzotriazol-2-yl)-4,6-ditertpentylphenol (UV-328) as a Substance of Very High Concern Because of Its PBT/vPvB-Properties. https://echa.europa.eu/documents/10162/78b46a52-7b7c-c7ae-d5d7-2df3d2ef3a21.

[56] Kim Y., Lee H., Jang M., Hong S.H., Kwon J.-H. (2021). Evaluating the fate of hexabromocyclododecanes in the coastal environment: Fugacity analysis using field data. Environ. Pollut..

